# Ecological and Evolutionary Processes Drive the Origin and Maintenance of Imperfect Mimicry

**DOI:** 10.1371/journal.pone.0061610

**Published:** 2013-04-12

**Authors:** Joseph S. Wilson, Joshua P. Jahner, Kevin A. Williams, Matthew L. Forister

**Affiliations:** 1 Department of Biology, Utah State University Tooele, Tooele, Utah, United States of America; 2 Department of Biology, University of Nevada Reno, Reno, Nevada, United States of America; 3 Department of Biology, Utah State University, Logan, Utah, United States of America; 4 Departamento de Zoologia, Universidade Federal do Paraná, Curitiba, Brazil; Consiglio Nazionale delle Ricerche (CNR), Italy

## Abstract

Although the forces behind the evolution of imperfect mimicry remain poorly studied, recent hypotheses suggest that relaxed selection on small-bodied individuals leads to imperfect mimicry. While evolutionary history undoubtedly affects the development of imperfect mimicry, ecological community context has largely been ignored and may be an important driver of imperfect mimicry. Here we investigate how evolutionary and ecological contexts might influence mimetic fidelity in Müllerian and Batesian mimicry systems. In Batesian hoverfly systems we find that body size is not a strong predictor of mimetic fidelity. However, in Müllerian velvet ants we find a weak positive relationship between body size and mimetic fidelity when evolutionary context is controlled for and a much stronger relationship between community diversity and mimetic fidelity. These results suggest that reduced selection on small-bodied individuals may not be a major driver of the evolution of imperfect mimicry and that other factors, such as ecological community context, should be considered when studying the evolution of imperfect mimicry.

## Introduction

Color mimicry is often celebrated as one of the most straightforward examples of evolution by natural selection, as striking morphological similarity between a mimic and its model evolves in response to predation pressure [1]. However, several examples of imperfect mimicry have also been documented [2]–[5]. For example, several species of hoverflies (Syrphidae) closely resemble bees or wasps, yet some species appear to be poor mimics, not matching the color patterns of any specific wasp or bee species [5]. A majority of studies investigating the evolution of imperfect mimicry have focused on Batesian mimicry (where a harmless mimic resembles a harmful model) [6]. Several hypotheses have been proposed regarding the evolution of imperfect Batesian mimicry, most of which focus on various conditions under which selection might be relaxed on mimics. While much has been written regarding selection and the evolution of mimicry, most hypotheses are centered on signal detection theory (e.g., [4], [7]–[10]), which explains how predators select for mimicry. Our purpose is not to review all of the alternative hypotheses regarding the evolution of imperfect mimicry, but to test some of those that have recently been proposed.

A recent study [5] tested several hypotheses regarding the evolution of imperfect mimicry using a hoverfly mimicry system (Diptera: Syrphidae) and found that of all the proposed hypotheses (including the eye of the beholder, multimodel, kin selection, constraints, and relaxed selection hypotheses), only one, which they call the relaxed selection hypothesis, was supported by their analysis. They found that small-bodied flies had lower mimetic fidelity, putatively driven by lowered selective pressure from predators that focus on larger-bodied prey [5]. While Penney et al. [5] refer to this as the relaxed-selection hypothesis, nearly all of the hypotheses on imperfect mimicry are based on various causes of relaxed selection. To distinguish the hypothesis dealing with relaxed selection due to small body size from other hypotheses regarding relaxed selection we will hereafter refer to the hypothesis proposed by Penney et al. [5] as the small bodied hypothesis. An alternative hypothesis has been proposed regarding the evolution of imperfect mimicry in Müllerian systems (where two or more species with effective secondary defenses share a similar appearance for mutual benefit) [11]. Ihalainen et al. [12] suggests that imperfect mimicry is more likely to evolve in diverse prey communities because prey are under relaxed selection due to increased generalization by predators [12]; for simplicity we refer to this as the community diversity hypothesis. Here, we investigate both the ecological context (community diversity) and evolutionary context (body size and mimicry ring) of multiple groups to understand the evolution of imperfect mimicry. While mimicry ring could be considered an ecological context, because each distinct ring is associated with contemporary and potentially-interacting species, here we consider mimicry ring an evolutionary context in reference to the action of natural selection shaping the phenotypes involved in the mimicry rings. In contrast to evolutionary history that has been more frequently studied, community diversity (what we refer to as ecological context) has rarely been considered in previous studies of mimicry [13]–[15] but has been shown to be important in driving other evolutionary processes [16], [17].

Understanding the drivers of imperfect mimicry has historically been problematic due to the challenge of quantifying mimetic fidelity (how closely a mimic resembles a model). While some researchers doubt the adequacy of human perception of mimetic fidelity, recent comparative analyses of various measures of mimetic fidelity have found that subjective human rankings [5], [18] are comparable to rankings based on multivariate analyses of morphological features [5], [19] and avian response rankings [20], [21] indicating that human rankings can be an effective measure of mimetic fidelity.

Although most of the studies on human perception of mimetic fidelity have investigated mimicry systems where birds are thought to be the primary predators [5], [18], [21], other potential predators like lizards have similar color vision to birds and humans [22] and likely perceive mimetic patterns in the same way.

The complex natures of Batesian and Müllerian mimicry systems, however, provide added difficulties in examining the evolution of imperfect mimicry. For example, in many cases there can be a strong geographic component to mimetic relationships, such that the ranges of potentially overlapping models and mimics must be considered. In addition, it can be especially difficult to effectively assay the match (or lack thereof) between mimics and models in groups where species richness in both models and mimics is high. For instance, hundreds of species of stinging Hymenoptera (bees, wasps, and ants) have been collected in a single location [23], many of which can be considered models in sympatric hoverfly mimetic systems, potentially causing measures of mimetic fidelity to differ depending on the model studied.

Here we address these complexities by studying unrelated Batesian and Müllerian mimetic systems within their respective ecological and evolutionary contexts, using human rankings of mimetic fidelity. First, we investigated the relationship between body size and mimetic fidelity in Batesian hoverfly mimics using methods and materials presented in a recent study by Penney et al. [5], who found that small body size predicted imperfect mimicry, though the authors did not consider geography in their analysis. Our objective in recreating the study of Penney et al. was to provide a point of comparison with analyses associated with our second dataset, while utilizing a common set of observers. Our second dataset consisted of images of Batesian hoverfly mimics and potential models, and similarly addressed the relationship between body size and mimetic fidelity, but using specimens collected from the same geographic region. Finally, we investigated the relationship between body size and mimetic fidelity in an exceptionally large Müllerian mimicry complex in North American velvet ants from the genus *Dasymutilla* (Hymenoptera: Mutillidae) [24]. In addition to addressing the possibility of relaxed selection associated with small body size in flies and velvet ants, we examined the effect prey community diversity has on the evolution of imperfect mimicry in velvet ants. Finally, we propose a variant of the community diversity hypothesis that may explain the evolution of imperfect mimicry in Batesian mimetic systems in which Hymenoptera are the models.

## Materials and Methods

### Mimetic fidelity

In order to repeat the study of Penney et al. [5], we showed photographs of the 38 mimics and 3 models available in their supplementary files to student volunteers (N = 41) following the protocol reported in Penney et al. [5]. This consisted of a photograph of each mimic shown on a slide presentation alongside the same images of a wasp (*Vespula vulgaris*), honeybee (*Apis mellifera*) and bumblebee (*Bombus impatiens*) for 20 seconds each. Volunteers were asked to rank each fly on a scale of 1 (very poor mimic) to 10 (excellent mimic) for each of the three potential models (wasp, honeybee and bumble bee). Each hoverfly and model image was presented at magnifications such that they had the same projected body length. The mimetic fidelity of each fly was estimated based on the highest mean score of a fly compared to any of the three models. In other words, each fly received three individual scores from each volunteer, one score comparing the fly to each model. The mean score (across students) was then calculated for a fly compared to each of the three models. The highest of these three means was considered the mimetic fidelity of that fly and was also used to identify which model it matched most closely.

#### Hoverfly dataset of Nevada specimens

To determine mimetic fidelity of flies compared to a more diverse and geographically-based model community, 10 fly species were compared to 10 hymenopteran models (Fig. S1) in a pairwise manner with a single fly being shown next to a single model. Flies were selected to represent the broad range of sizes found within Syrphidae and models were selected in order to represent the broad morphological diversity within Hymenoptera. All specimens were taken from the Nevada State Entomology Collection in Reno, Nevada. While exact collection localities were not available for all specimens (i.e., some specimens have only vague locality information like “Washoe County Nevada”), an effort was made to only use mimics and models that would potentially co-occur. A randomized slideshow containing all of the 100 possible fly-model combinations was presented to volunteers (N = 54) who were directed to rank each fly on a scale of 1 (very poor mimic) to 10 (excellent mimic) compared to the model it was paired to. Each slide was presented for 10 seconds. Each hoverfly and model image were presented at magnifications such that they had the same projected body length. The mimetic fidelity of each fly was estimated based on the highest mean score of a fly compared to any of the 10 models.

#### Velvet ant dataset

To measure mimetic fidelity of velvet ants involved in described Müllerian mimicry rings [24] we selected five members of each ring to represent the range of sizes of individuals in the ring (Table 1). Because mimetic fidelity in Müllerian systems represents how well a given species mimics a group of species (i.e., the mimicry ring), we presented slides showing an individual species (Table 1) compared to all of the other members of the mimicry ring that the species was assigned to [24]. While some phenotypic variation exists within many species, primarily in the shade of the colored setae, individuals used in comparisons were selected because they represented a typical phenotype based on examination of hundreds of specimens of each species from insect museums across North America. Each slide was presented for 20 seconds. Volunteers (N = 113) were directed to rank each velvet ant on how well it seemed to fit into the mimicry ring it was shown with. Rankings were based on a scale of 1 (very poor mimic) to 10 (excellent mimic). All velvet ant images were presented at magnifications such that they had the same projected body length. The mimetic fidelity of each velvet ant was estimated based on the mean score of a velvet ant compared to its assigned mimicry ring.

**Table 1:** Velvet ant species used in the analysis and the size (mm) of each specimen. Species are grouped by mimicry ring.

Madrean ring	Size	Desert ring	Size	Texan ring	Size	Tropical ring	Size	Eastern ring	Size	Western ring	Size
*D. sicheliana*	15	*D. magna*	22	*D. klugii*	22	*D. pulchra*	19	*D. occidentalis*	20	*D. calorata*	22
*D. citromaculosa*	14	*D. nocturna*	17	*D. biocculata*	12	*D. cressoni*	18	*D. biocculata*	13	*D. vestita*	13
*D. ferruginea*	10	*D. gloriosa*	13	*D. wileyae*	11	*D. arachnoides*	13	*D. quadriguttata*	12	*D. coccineohirta*	11
*D. dilucida*	9	*D. pseudopappus*	12	*D. zelaya*	10	*D. zoster*	10	*D. scaevola*	12	*D. californica*	7
*D. asteria*	6	*D. thetis*	7.5	*D. nupera*	7	*D. spilota*	6	*D. canella*	6	*D. atricauda*	7

All volunteers participating in this study were students majoring in Biology or related disciplines at the University of Nevada, Reno. Volunteers were recruited from an upper division ecology course and from an introductory biology course. Students in each course were presented with a short presentation introducing the concepts of Batesian and Müllerian mimicry and were then given the option to participate in a survey designed to rank mimetic fidelity of various insects. If students agreed to participate, they were presented with a score sheet and were invited to view the presentation described above. To our knowledge, the volunteers were not experts in insect identification. This effectively resulted in mimetic fidelity scores that were based on overall resemblance of a mimic to a model rather than on preconceived ideas of what specific parts of a mimic should match a model. None of the volunteers were minors (i.e., all participants were over the age of 18) and no data relating to the volunteers were gathered, so there was no need to anonymize. After explaining the research to the Office of Human Research at the University of Nevada, Reno it was concluded that an official institutional review board (IRB) review of the research was not needed and no official waiver would be necessary. Therefore, No IRB approval was requested for this research because no information about living individuals was collected, meaning the research does not involve human subjects as per the Code of Federal Regulations 45 CFR part 46. Volunteers were simply used to gather information (in this case morphological similarities) about the insects involved in this study. Because of the need to protect the anonymity of our volunteers, no questions were asked regarding any physical characteristics that would make ranking mimics and models difficult (i.e., colorblindness). While this potentially could affect the reported mimetic fidelity scores, we feel any influence of colorblindness would be minimal largely due to the aposematic signals in hoverflies and their models as well as velvet ants. These warning signals primarily result from contrasting black and red or yellow patterns, which would still be visually distinct to colorblind individuals.

### Body size

While thorax length is often used in studies of insect size, Penney et al. [5] used a principle component analysis of antenna length, abdomen width and length, thorax width, wing length, and head width to measure body size. Because their dataset did not include thorax length we tested the correlation between various individual measures to their measure of body size (PC1). We found that both thorax width and abdomen length were highly correlated to PC1 (r = −0.928). For ease of measurement, body size measurements of the flies used in this study were based on abdominal length, which is highly correlated to, and provides the same relationship between size and mimetic fidelity as other body size measures used by Penney et al. [5]. Velvet ant body sizes consisted of the entire length of the body of each species. All measurements were taken on individual specimens and were measured with a transparent ruler to the closest millimeter.

### Velvet ant community diversity

While the community diversity hypothesis is based on prey community diversity, we suggest that the proposed relaxed selection in more diverse communities would be a result of morphological diversity rather than species diversity. For example, if five velvet ant species were present in an area and all five were high fidelity mimics of each other, there would be relatively less selective pressure for increased generalization by predators. Alternatively, if there were five velvet ant species in an area, but each species each participated in a different mimicry ring, there would be increased selective pressure for generalization by predators. For this reason we focus on morphological community diversity rather than species diversity.

Community diversity of each velvet ant mimicry ring was estimated based on a Shannon diversity index [25]. Because actual species diversity and individual abundance data are not known and because of the reasons outlined above, a proxy was used to calculate the diversity indices for each mimicry ring. This proxy was calculated by measuring the amount of geographic overlap in each mimicry ring based on the map presented by Wilson et al. [24] (Fig. S2). The number of overlapping mimicry rings was used as a proxy for morphological species richness and the amount of overlap was used as a proxy for morphological species abundance. All measurements were made using ImageJ (http://rsb.info.nih.gov/ij/). Diversity measures for each mimicry ring were as follows: Madrean = 1.134, Desert = 0.893, Texan = 0.761, Western = 0.505, Eastern = 0.086, Tropical = 0.062. These diversity measures are similar to unquantified field observations in that areas with large overlaps in multiple mimicry rings as presented in figure S2 (e.g., southeastern Arizona) also have high morphological diversity in velvet ants and areas with little overlap in mimicry rings (e.g., Georgia) have low morphological diversity in velvet ants.

### Analyses

To determine if our survey methodology resulted in the same relationship presented by Penney et al. [5], we used a linear regression with hoverfly size as the predictor variable and mimetic fidelity as the response variable. To determine if the comparison of mimics to a more diverse and geographically-selected model community had the same relationship as found in Penney et al. [5], we used an analysis of covariance (ANCOVA) with mimetic fidelity as the response variable and body size, dataset, and the interaction between body size and dataset as predictor variables. To control for the phylogenetic independence of the velvet ant data, we calculated phylogenetic independent contrasts for body size and mimetic fidelity using the APE package in R [26], [27]. Phylogenetic independent contrasts were not calculated for the hoverfly data because Penney et al. [5] found no relationship between phylogeny and mimetic fidelity in hoverflies. For the calculation of independent contrasts we used the molecular-based phylogenetic tree presented by Wilson et al. [24]. To test the small bodied hypothesis in velvet ants, we used a linear regression with mimic size as the predictor variable and mimetic fidelity as the response variable. The linear regression between body size and mimetic fidelity was repeated using phylogenetically corrected data (see phylogenetic independent contrasts above) to control for the phylogenetic independence of the data. To investigate whether or not evolutionary context (i.e. mimicry ring) affected the relationship between body size and mimetic fidelity, we used an ANCOVA with mimetic fidelity as the response variable and body size, mimicry ring, and the interaction between body size and mimicry ring as the predictor variables (this model and subsequent models were not phylogenetically corrected because mimicry ring is not a relevant variable following phylogenetic correction). To test the community diversity hypothesis in velvet ants, we used a linear regression with average mimetic fidelity for each mimicry ring as the response variable and the Shannon diversity index as the predictor variable. All analyses were performed in Program R 2.11.1 [28] and all ANCOVA's were performed in the *car* package [29] using type-III tests.

## Results

**Figure 1:**
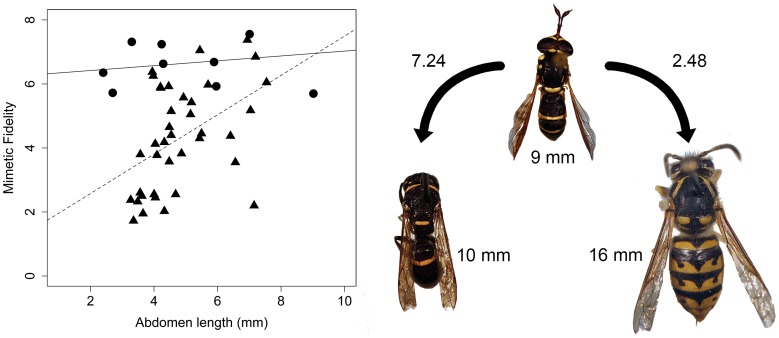
Relationships between body size and mimetic fidelity in hoverflies. Triangles represent reanalyzed mimics from Penney et al. [5] and circles indicate mimics compared to potential models from Nevada. The dashed line shows the linear regression of the reanalyzed data and the solid line shows the linear regression of the data with the Nevada dataset. Also shown are the mimetic fidelity scores between a mimetic fly and a similar sized model (Eumenidae) and a larger sized model (Vespidae). Insects are depicted approximately to scale.

Our re-investigation of the models and mimics presented by Penney et al. [5] using 38 fly species and three models (honey bee, bumble bee, yellow jacket) provided results that were qualitatively identical to the original study, namely a positive relationship between size and mimetic fidelity (*R* ^2^ = 0.21, *P* = 0.004; Fig. 1). However, when we performed a similar experiment using a more diverse and geographically coextensive model community, comparing 10 fly mimics with 10 sympatric hymenopteran models (supplementary Fig. 1), we found no relationship between body size and mimetic fidelity (*F*_1,44_ = 0.22, *P* = 0.639; Fig. 1). The difference between our results and the results of Penney et al. [5] is demonstrated by a significant interaction (*F*_1,44_ = 4.82; *P* = 0.033) between dataset (as a variable in analysis of covariance) and mimic size (Fig. 1). When compared against sympatric mimics with which they potentially share an ecological and evolutionary history, the mimics in our experiment received uniformly high mimetic fidelity scores (Fig. 1), contrary to the small bodied hypothesis.

**Figure 2:**
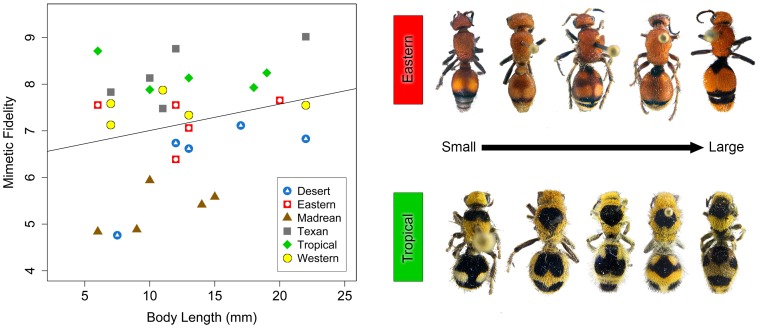
Relationship between body size and mimetic fidelity in velvet ants. Line illustrates the relationship between mimetic fidelity and body size, which is significant when accounting for mimicry ring. Although an effect of mimicry ring on mimetic fidelity was detected (see main text), a single regression line across all rings is shown for ease of visualization. Also shown are five individuals from two mimicry rings showing the morphological variation within each ring. Individuals are not depicted to scale.

We then performed an additional test of the small bodied hypothesis using individuals from six velvet ant Müllerian mimicry rings [24]. Initially, we did not find evidence for a significant relationship between body size and mimetic fidelity (*R* ^2^ = 0.06, *P* = 0.20), similar to the results from our investigation into the Batesian hoverfly system when considering sympatric models and mimics. The linear regression between body size and mimetic fidelity was also non-significant when using phylogenetically corrected data (*F*_1,27_ = 0.354; *R^2^* = 0.01; *P* = 0.557). We can use information related to the evolutionary context of the velvet ants, however, by accounting for variation among mimicry rings (as a categorical variable) in mimetic scores. When we statistically accounted for variation among rings in mimetic fidelity, we found significant effects of body size (*F*_1,18_ = 7.59, *P* = 0.013; Fig. 2) and mimicry ring (*F*_5,18_ = 5.55, *P* = 0.003; Fig. 2) and a non-significant interaction between body size and mimicry ring (*F*_5,18_ = 1.59, *P* = 0.215). While there is a weak relationship between body size and mimetic fidelity in the velvet ants, mimicry ring has a much stronger affect on mimetic fidelity than body size.

**Figure 3:**
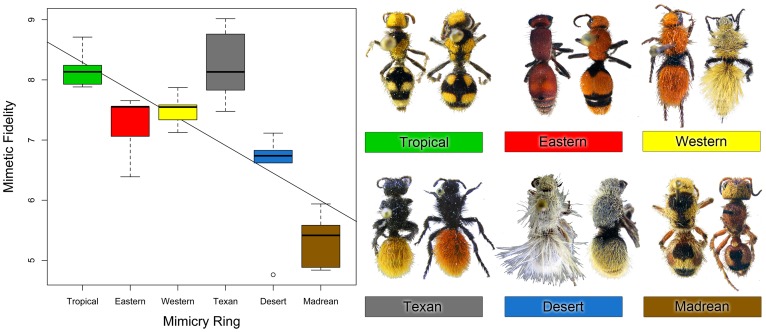
Mimetic fidelity found in each velvet ant mimicry ring. Boxplots of mimetic fidelity are shown for each mimicry ring with outliers shown as open circles. Mimicry rings are ordered by increasing community diversity scores along the x-axis (i.e. highest diversity in the Madrean ring). Best-fit line is shown; for ease of visualization the line is based on ranked diversity indices (full analyses are based on raw diversity values, see Methods). Also shown are examples of morphological diversity in each mimicry ring.

Unlike the small bodied hypothesis, the community diversity hypothesis, which predicts relaxed selection on mimicry in systems where predators interact with a large suite of prey, has not been tested in a natural system. Each of the six velvet ant mimicry rings differ in prey community diversity. The prey community in this sense is the group of geographically overlapping mimicry rings: i.e. high diversity corresponds to a geographic region in which potential velvet ant predators interact with species from multiple, morphologically-divergent mimicry rings. The Madrean and Desert mimicry rings have the highest community diversity while the Tropical and Eastern rings have the lowest (Fig. 3). Prey community diversity was negatively correlated with mimetic fidelity in velvet ants (*R* ^2^ = 0.36, *P*<0.001; Fig. 3), consistent with the community diversity hypothesis.

## Discussion

### Small bodied hypothesis

Our results do not find any support for the small bodied hypothesis in hoverflies. Instead we find uniformly high mimetic fidelity among small and large flies. We suggest that the support for this hypothesis found in other studies [5] was likely a result of an experimental design that did not account for the diverse suite of potential models that exist in nature. Although our data suggest that local effects may play a strong role in the development of high fidelity mimics (i.e., mimetic species might be higher fidelity mimics to sympatric models than they are to allopatric models), this was not directly testable with our data because, like many insects, the ranges of each species used in our analysis is only poorly known so no comparisons of sympatry vs mimetic fidelity were possible. Furthermore, our dataset of Nevada mimics and models is not exhaustive and many potential models exist for each of the fly species we used in our test. For example, the small (4 mm) fly with a red abdomen used in our analysis (Fig. S1) was found to be a relatively high fidelity mimic to a small sphecid wasp (Fig. S1). In addition to this wasp, there are dozens of other similarly sized wasp and bee species with similar coloration, which would likely also be considered good models for this small fly. Future analyses should be designed to specifically test the effect of amount of sympatry of models and mimics on mimetic fidelity.

While we did not find evidence supporting the small bodied hypothesis in hoverflies, our results lend some support to this hypothesis in the velvet ant mimicry system, where we found a positive relationship between body size and mimetic fidelity, but only when evolutionary context (mimicry ring) is included in the statistical model (Fig. 2). In many of the mimicry rings there is no clear relationship between mimetic fidelity and size (e.g., the Western, Tropical, Texan, and Eastern mimicry rings, Fig. 2), yet in others there is a suggestion that smaller specimens are lower fidelity mimics (e.g., Desert mimicry ring, Fig. 2). Because our study was not designed to intensively sample within mimicry rings (only five specimens from each mimicry ring were analyzed), it is possible that future analyses including more specimens will result in more definitive conclusions.

### Community diversity hypothesis

Unlike body size, ecological context (community diversity) appears to play a strong role in the evolution and maintenance of imperfect mimicry, as proposed through the community diversity hypothesis. In the velvet ant mimicry system, we suggest that community complexity could be a function of how many different color forms are present in any velvet ant community. Southern Arizona and Northwestern Mexico house the most diverse velvet ant communities in North America in terms of species diversity [30] and morphological diversity (i.e., diversity of distinct mimicry rings in a given area) [24]. Members of the Madrean mimicry ring, which have the lowest average mimetic fidelity scores (Fig. 3), are most abundant in these areas. This suggests that high morphological diversity in the velvet ant community might lead to lower mimetic fidelity, perhaps due to reduced selection for perfect mimicry. It should be considered, however, that morphological community diversity as presented here is only a rough estimate of true morphological diversity in velvet ants. This analysis only considers the morphological diversity in the described mimicry rings in one genus, *Dasymutilla*. There are dozens of other velvet ant genera in North America that potentially participate in mimicry rings (Pers. Obs.). These other genera could influence the true morphological community diversity and may explain why members of some of the rings included in this analysis that received low diversity scores (e.g., Eastern = 0.086, Tropical = 0.062) did not all receive high mimetic fidelity scores. While our results clearly support the community diversity hypothesis, additional analyses will be needed in order to test all the complexities involved in the evolution of mimetic fidelity in velvet ants.

Our analyses find that mimicry ring is a strong predictor of mimetic fidelity, which we have linked to community diversity. A potentially confounding factor in this assertion is geography. By definition, species involved in mimicry rings are geographically concordant, with the majority of species involved in each mimicry ring living in a defined geographic area [24]. Because of this, it is difficult to determine if the community morphological diversity is driving imperfect mimicry or if the local differences in environmental parameters within each mimicry ring's geographic range are affecting mimetic fidelity. Recent advances in ecological niche modeling should enable future tests of this by measuring the bioclimatic variables that affect the range of each mimicry ring and comparing these variables to measures of mimetic fidelity. At this point, most velvet ant collection localities have not been databased, making the construction of ecological niche models impossible until such databases are constructed.

The community diversity hypothesis, while developed based on a hypothetical Müllerian system and supported by our analysis of the Müllerian velvet ant system, might also apply to Batesian systems [12]. Predators foraging in a diverse community may learn to generalize, therefore lowering selection for perfect Batesian mimics [12]. While community diversity was not measured in our analyses of Batesian systems, we propose a new hypothesis (the sociality hypothesis) that is complementary to the community diversity hypothesis to predict mimetic fidelity in hoverflies. The sociality hypothesis states that mimetic fidelity is positively correlated with the model's degree of sociality because the higher population abundances and low intraspecific morphological variation in social hymenoptera effectively results in a morphologically simple prey community. While our analyses did include social and solitary model species, only three social species and seven solitary species were included, which is too small of a dataset to effectively test the sociality hypothesis. Similarly, all of the models used by Penney et al. [5] were social species, making their dataset unsuited to testing the sociality hypothesis as well.

### Summary

Our analyses suggest a complex mixture of evolutionary and ecological context influences the evolution of imperfect mimicry. We find that community diversity is among the strongest predictors of mimetic fidelity in Müllerian mimetic systems, and we propose hypotheses that will hopefully be useful in future research in the area of imperfect mimicry. These findings are consistent with a growing body of work highlighting the need to consider interactions among ecological and evolutionary processes [16], even when studying phenomena that seem superficially to be straightforward examples of natural selection.

## Supporting Information

Figure S1 **Images of the 10 hoverfly mimics (top row) and 10 hymenopteran models (bottom row). Body sizes are given for each insect.**
(TIF)

Figure S2 **Map of the six velvet ant mimicry rings as presented by Wilson et al. (2012).**
(TIF)
