# Supplementary figures and images for: Ecological and Evolutionary Processes Drive the Origin and Maintenance of Imperfect Mimicry
Source: PLoS One. 2013 Apr 12;8(4):e61610. doi: 10.1371/journal.pone.0061610 (PMC3625143; doi:10.1371/journal.pone.0061610)

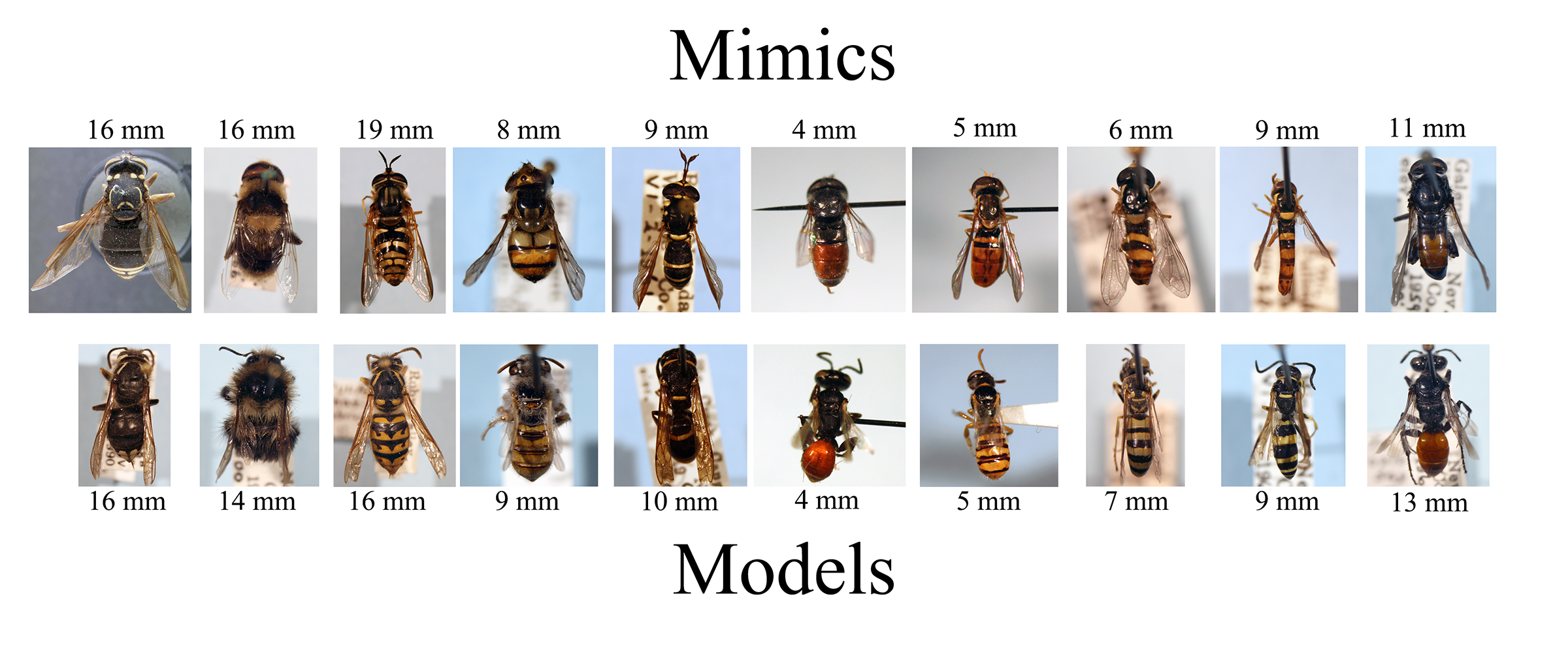

Supplement: Figure S1 — Images of the 10 hoverfly mimics (top row) and 10 hymenopteran models (bottom row). Body sizes are given for each insect. (TIF) [file pone.0061610.s001.tif]

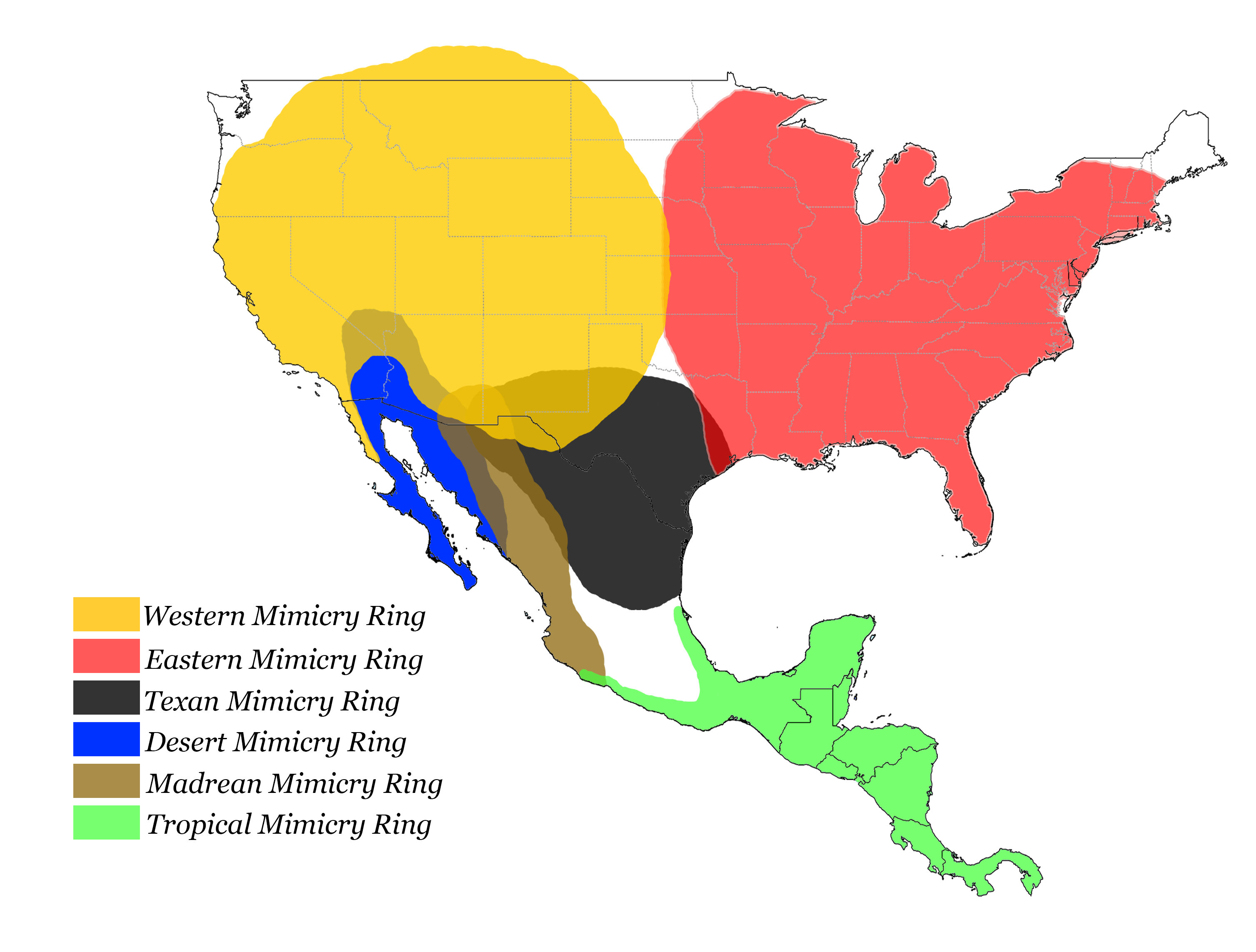

Supplement: Figure S2 — Map of the six velvet ant mimicry rings as presented by Wilson et al. (2012). (TIF) [file pone.0061610.s002.tif]
